# Supplementary material for: Cardiac Molecular Analysis Reveals Aging‐Associated Metabolic Alterations Promoting Glycosaminoglycans Accumulation via Hexosamine Biosynthetic Pathway
Source: Adv Sci (Weinh). 2024 Aug 9;11(38):2309211. doi: 10.1002/advs.202309211 (PMC11481188; doi:10.1002/advs.202309211)
Supplement: Supplementary file 1 — Supporting Information [file ADVS-11-2309211-s001.docx]

**Figure S1**


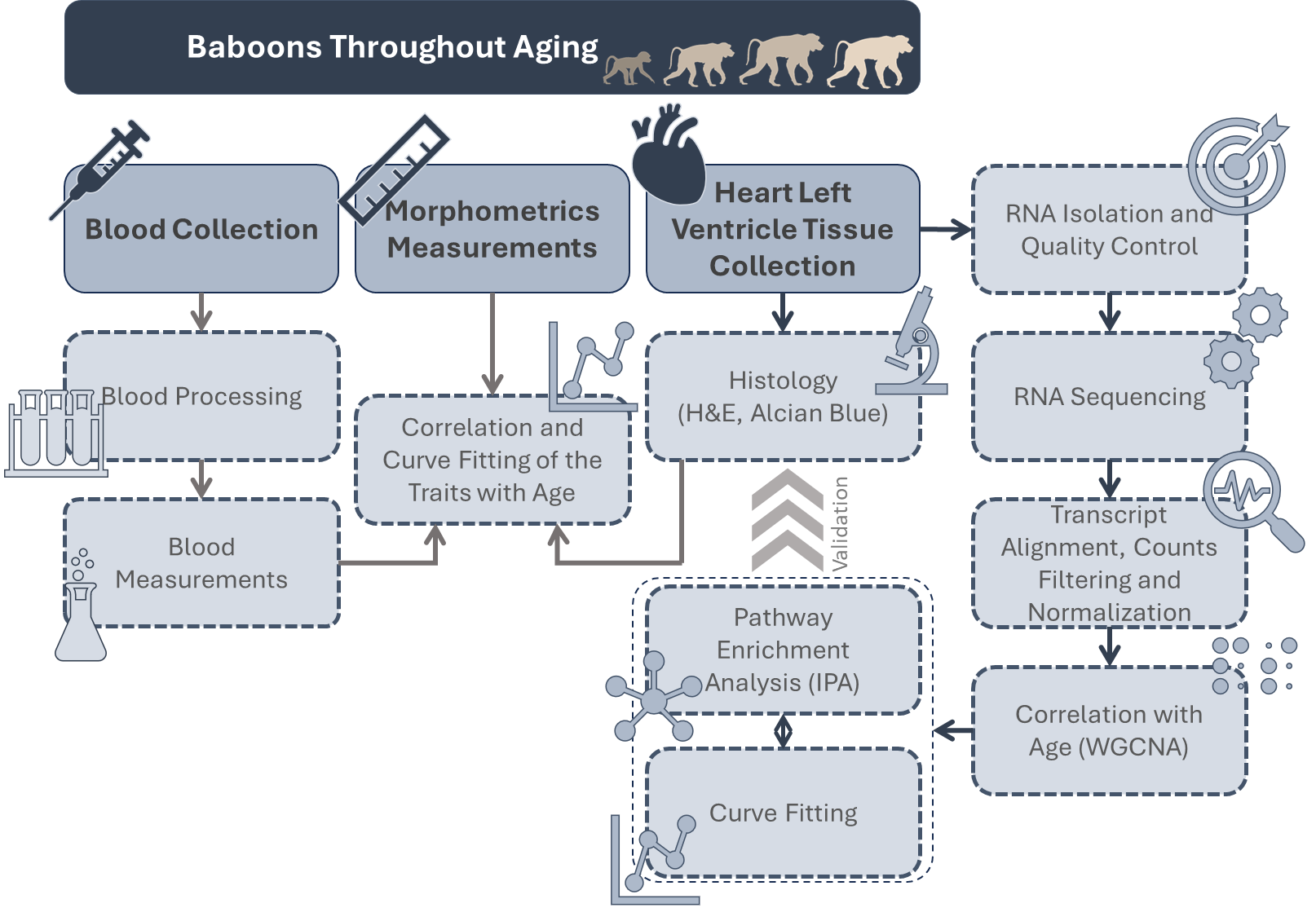


**Figure S1 – Schematic representation of the experimental approach used in this manuscript.** Baboons aged from 7.5 to 22.1 years old and blood was collected, morphometric measurements obtained and heart left ventricle tissue was collected at necropsy for each animal. Morphometrics, blood, and heart histology measurements were correlated with age and the proper curve fitting was evaluated. Heart left ventricle tissue was used to isolate RNA followed by RNA Sequencing, data treatment, and associated with age using WGCNA. Transcripts identified were used for the network and pathway enrichment analysis and for curve fitting. The major findings were validated histologically.

**Figure S2**


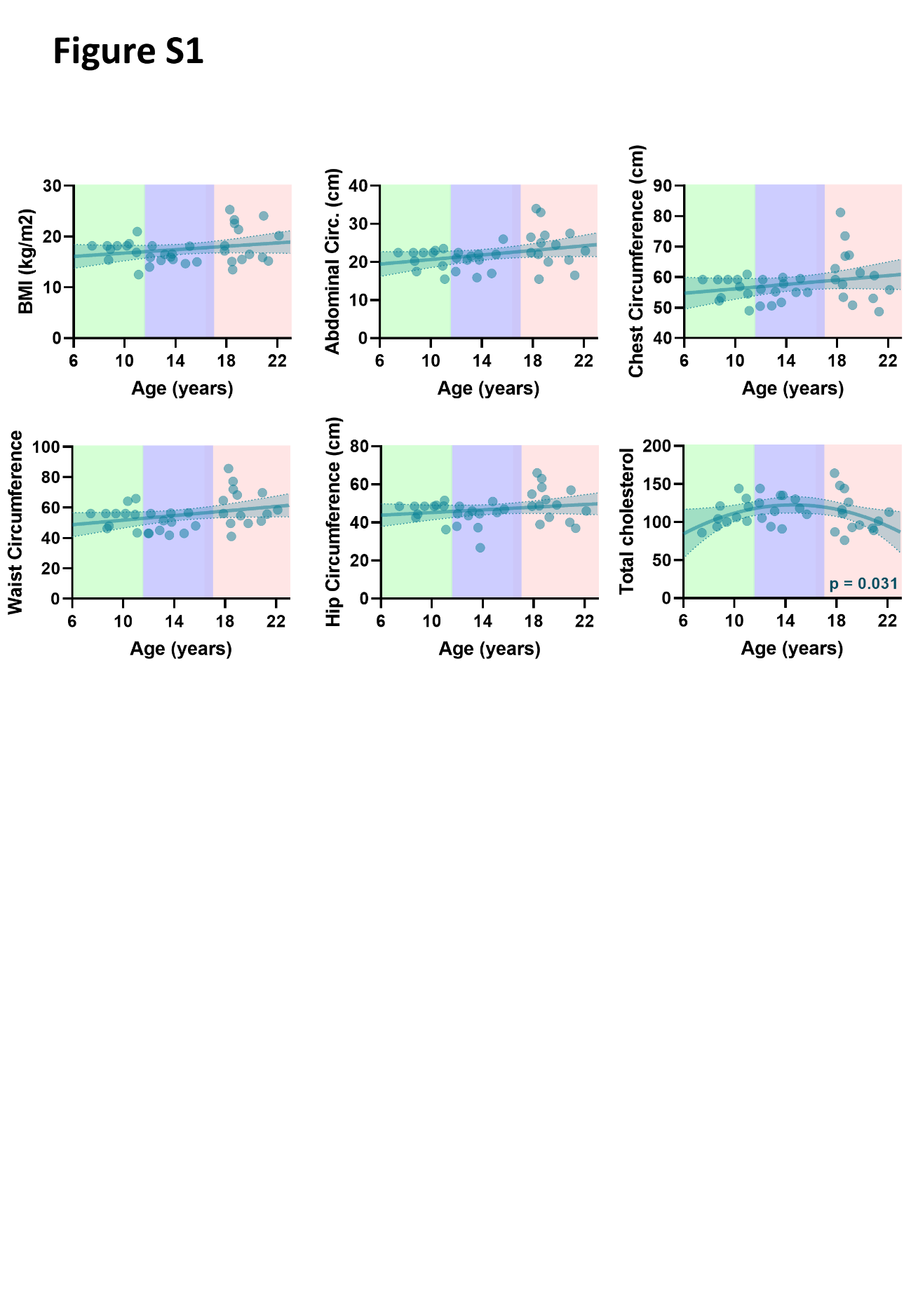


**Figure S2 - Impact of aging on baboon morphometrics and blood clinical data.** Variation of morphometric (BMI, abdominal, chest, waist, and hip circumference) and blood clinical (total cholesterol) measurements with age. Circ. – circumference.

**Figure S3**


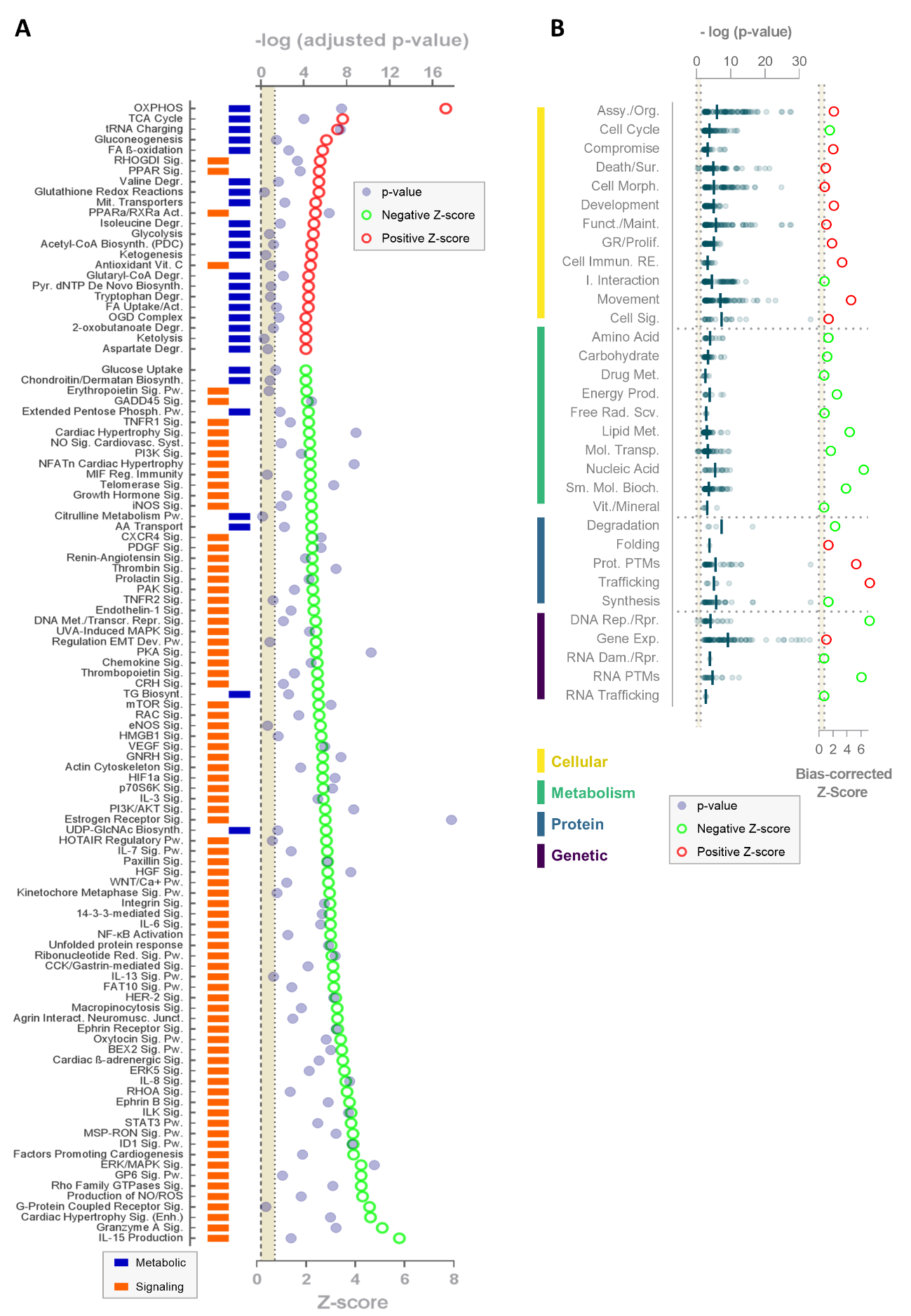


**Figure S3 - Cardiac age-related transcriptomic pathway enrichment analysis in NHP females.** (A) Comparison of canonical pathways predicted activation and inhibition (absolute z-score≥2) with age. Positive z-scores are represented in red, negative z-scores in green, and the p-value in blue. The color depicts metabolic (blue) or signaling (orange) canonical pathways. (B) Biofunctions identified for the pathway enrichment analysis. Each dot represents the -log(p-value) of a biofunction. The color represents cellular (in yellow), metabolic (in green), protein-related (in blue), and genomic (in purple) biofunctions. Each circle represents the bias-corrected z-score and the respective color a negative, in green, or a positive, in red, directionality. Act. – activation; Assy. – assembly; Bioch. – biochemical; Biosynth. – biosynthesis; Dam. – damage; Degr. – degradation; Enh. – enhanced; Exp. – expression; FA – fatty acid; Funct. – function; GR – growth; Immun. – immunity; Interact. – interaction; Junct. – junction; Maint. – maintenance; Met. – metabolism; Met. – methylation; Mol. – molecular; Morph. – morphology; Neuromus. – neuromuscular; Org. – organization; OXPHOS – oxidative phosphorylation; Phosph. – phosphate; Prod. – production; Prolif. – proliferation; Prot. PTMs – protein post-translational modifications; Pw. – pathway; Rad. – radical; RE. – response; Red. – reductase; Reg. – regulatory; Rep. – replication; Repr. – Repression; RNA PTMs – RNA post-transcriptional modifications; Rpr. – repair; Scv. – scavenger; Sig. – signaling; Sm. – small; Sur. – survival; Syst. – system; TCA - tricarboxylic acid; Transcr. – transcriptional; Transp. – transport; Vit. – vitamin.

**Figure S4**


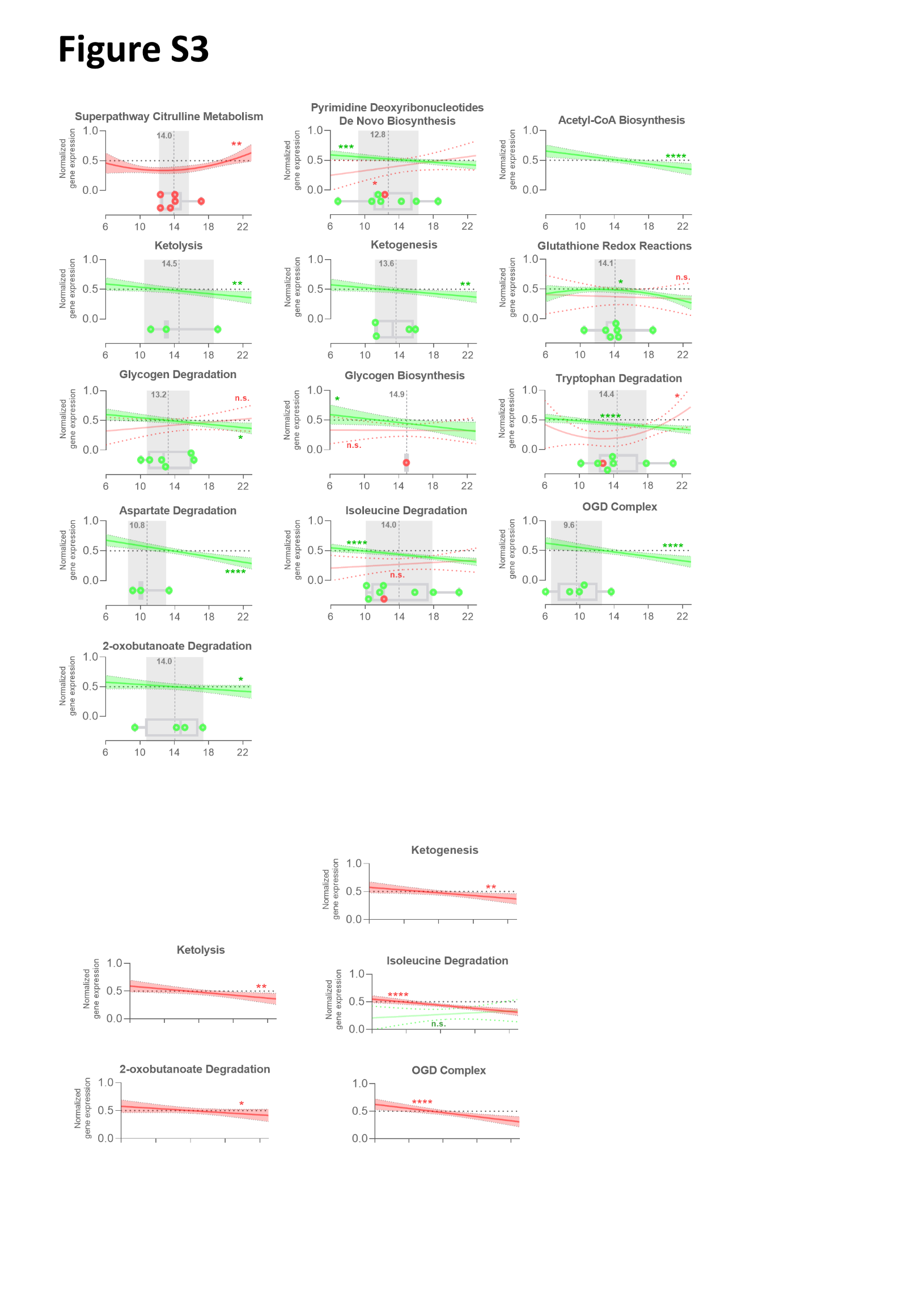


**Figure S4 – Longitudinal gene expression of pathways involved in metabolism in the NHP female heart.** Longitudinal profile of relative transcript expression with age, boxplot with transcripts tipping points (red – upregulated, green – downregulated), and the age of the average tipping point (grey) from the metabolic canonical pathway.
